# Integrating phenotype ontologies with PhenomeNET

**DOI:** 10.1186/s13326-017-0167-4

**Published:** 2017-12-19

**Authors:** Miguel Ángel Rodríguez-García, Georgios V. Gkoutos, Paul N. Schofield, Robert Hoehndorf

**Affiliations:** 1 Computational Bioscience Research Center (CBRC), King Abdullah University of Science and Technology, 4700 KAUST, Thuwal, 23955-6900 Saudi Arabia; 2 Computer, Electrical and Mathematical Sciences & Engineering Division (CEMSE), King Abdullah University of Science and Technology, 4700 KAUST, PO Box 2882, Thuwal, 23955-6900 Saudi Arabia; 3 College of Medical and Dental Sciences, Institute of Cancer and Genomic Sciences, Centre for Computational Biology, University of Birmingham, Birmingham, B15 2TT UK; 4 Institute of Translational Medicine, University Hospitals Birmingham, NHS Foundation Trust, Birmingham, B15 2TT UK; 5 Institute of Biological, Environmental and Rural Sciences, Aberystwyth University, Aberystwyth, SY23 2AX UK; 6 Department of Physiology, Development & Neuroscience, University of Cambridge, Downing Street, Cambridge, CB2 3EG UK

**Keywords:** Phenotype, PhenomeNET, Disease gene prioritization, OWL, Automated reasoning

## Abstract

**Background:**

Integration and analysis of phenotype data from humans and model organisms is a key challenge in building our understanding of normal biology and pathophysiology. However, the range of phenotypes and anatomical details being captured in clinical and model organism databases presents complex problems when attempting to match classes across species and across phenotypes as diverse as behaviour and neoplasia. We have previously developed PhenomeNET, a system for disease gene prioritization that includes as one of its components an ontology designed to integrate phenotype ontologies. While not applicable to matching arbitrary ontologies, PhenomeNET can be used to identify related phenotypes in different species, including human, mouse, zebrafish, nematode worm, fruit fly, and yeast.

**Results:**

Here, we apply the PhenomeNET to identify related classes from two phenotype and two disease ontologies using automated reasoning. We demonstrate that we can identify a large number of mappings, some of which require automated reasoning and cannot easily be identified through lexical approaches alone. Combining automated reasoning with lexical matching further improves results in aligning ontologies.

**Conclusions:**

PhenomeNET can be used to align and integrate phenotype ontologies. The results can be utilized for biomedical analyses in which phenomena observed in model organisms are used to identify causative genes and mutations underlying human disease.

**Electronic supplementary material:**

The online version of this article (doi:10.1186/s13326-017-0167-4) contains supplementary material, which is available to authorized users.

## Background

Understanding the functions of genes and gene products is vital for our understanding of normal biology and pathophysiology. In recent years the amount of genotype and phenotype data available for species as distinct as man and model organisms such as nematode worms has increased dramatically and continues to accelerate. Insights from non-human species have an important role to play in our understanding of human biology [1] and the challenge is to mobilise this data in a way in which it can be used to give meaningful insights into human physiology and disease. While much data is now being captured formally using ontologies, data integration and comparison across species presents a major informatics challenge [2]. This task requires that related phenotypes which span levels of granularity as well as domains of knowledge, for example behaviour or neoplasia, in organisms as anatomically distinct as zebrafish and man, can be matched and compared so as to allow findings in one species to be related to others.

In response to this challenge we developed PhenomeNET. PhenomeNET [3] was built in 2011 as a system for disease gene discovery and prioritization. PhenomeNET consists of an ontology integrating species-specific phenotype ontologies based on the PATO ontology [4] and relations between anatomical structures and physiological processes, a database of gene-to-phenotype associations, and a measure of similarity between sets of phenotypes. Within PhenomeNET, species-specific phenotype ontologies are combined so that phenotypes observed in different species can be compared directly. The main application of PhenomeNET is the prioritization of candidate genes for human diseases by comparing human disease phenotypes to existing gene-phenotype associations derived from model organisms. In particular, human phenotypes associated with a disease can be compared to phenotypes observed in mouse or other model organisms using the integrated PhenomeNET ontology, and similarity between phenotypes can then be used to indicate the genetic basis of a disease. PhenomeNET has been successfully used to find candidate genes for diseases [3, 5], identify novel pathways [6], and repurpose drugs using mouse model phenotypes [7, 8].

The PhenomeNET ontology was originally built by formally integrating species-specific phenotype ontologies, permitting the relationship between classes of different phenotype ontologies to be determined through deductive inference. For this purpose, PhenomeNET relies on the UBERON [9] ontology that identifies equivalences between anatomy ontologies of different species, the Gene Ontology (GO) [10] as a means to identify equivalent or related processes and functions, and the PATO ontology [11] to identify the qualities associated with anatomical entities or biological processes.

Here, we use the PhenomeNET ontology to identify alignments between phenotypes in different species. We present our results based on three versions of the PhenomeNET ontology: the first version consists of the plain ontology using only the axioms provided in the Human Phenotype Ontology (HPO) [12] and the Mammalian Phenotype Ontology (MP) [13]; in the second version, we extend our original ontology by adding additional lexical and structural mappings generated with the AgreementMakerLight [14] system and represent them as equivalent class axioms in our ontology; and in the third version, we further generate mappings between classes in the PhenomeNET ontology, the Disease Ontology (DO) [15] and the Orphanet Rare Disease Ontology (ORDO) [16].

We find that our axiomatic approach can identify a large number of relations between classes that are not currently identified by other systems that do not utilize similar formal methods. However, our evaluation also shows that a large number of mappings can still be identified through lexical and structural approaches, and that a purely axiomatic approach will miss many mappings that cannot currently be identified axiomatically due to incomplete and underspecified formalization of phenotype classes. We illustrate how a combination of formal, lexical and structural approaches generates the most complete and comprehensive mappings between (phenotype) ontologies, and these mappings improve the application of phenotype ontologies in data analysis and translational research.

## Methods

### Data sources and ontologies

In our experiments, we use the Human Phenotype Ontology (HPO) [12], Mammalian Phenotype Ontology (MP) [13], Human Disease Ontology (DO) [15], and Orphanet Rare Disease Ontology (ORDO) [17] provided as part of the Ontology Alignment Evaluation Initiative 2016 competition.

The HPO is an ontology of human phenotypes and consists of 11,787 classes that provide a standarized vocabulary for describing phenotypic abnormalities which have been commonly encountered in human monogenic diseases [18]. The MP is mainly used to characterize mouse phenotypes, but can also be applied to other organisms. It consists of 11,720 classes that have been organized into a directed acyclic graph (DAG) and can be used to describe abnormal phenotypes of physiological and anatomical systems, behavior, and survival [19].

DO provides a classification of human diseases according to multiple axes related to genetic disorders, infectious diseases, metabolic disorders. It consists 9247 classes that aim at unifying the representation of human diseases defined across a variety of developed biomedical vocabularies [20].

ORDO is derived from the Orphanet database of rare and orphan diseases and used to represent and categorize the diseases within Orphanet. It consists of 12,960 classes which provides a structured vocabulary to represent relationships between phenomes, diseases, genes and relevant features such genetic inheritance for analyzing rare diseases [17].

### Lexical mappings

We use the AgreementMakerLight (AML) [21], released on 5 April 2016, to generate lexical mappings between ontologies. We used the automatic match mode of the AML with the default settings to generate the lexical mappings that were used to extend the PhenomeNet ontology. The default settings of AML include use of the UBERON ontology, DO, and Wordnet as background knowledge, a lexical matcher, a word-based matcher that evaluates occurrence of the same words in class labels and synomyms, and a string similarity measure (ISub).

In addition to mappings generated by the AML, we also incorporate mappings between the ontologies obtained from BioPortal [22]. For each mapping between classes from two ontologies, we add an equivalent class axiom to the PhenomeNET ontology.

### Semantic similarity and evaluation data

For additional external evaluation of our generated mappings, we apply the PhenomeNET ontology to the prioritization of candidate genes of human disease [3]. We use the phenotypes associated with knockout mice available from the Mouse Genome Informatics (MGI) database [23] and the phenotypes associated with human diseases from the Human Phenotype Ontology database [12]. We apply Resnik’s semantic similarity measure [24] together with the Best Matching Average strategy [25] to combine class similarities.

We evaluate the results using a list of gene–disease associations provided by the Human Phenotype Ontology database [12] as well as a set of mouse models of human disease provided by the MGI database [23].

### Source code and experiments

Source code for the PhenomeNET matching system, including parameter files, and the generated alignments, are available at http://github.com/bio-ontology-research-group/OAEI2016. Code to generate the PhenomeNET ontology is available at https://github.com/bio-ontology-research-group/phenomeblast/tree/master/fixphenotypes.

## Results

### Combining knowledge-based and lexical approaches for ontology integration

We developed and extended the PhenomeNET ontology to integrate several species-specific phenotype ontologies and identify mappings between phenotype classes. Here, we consider a mapping between two classes (in two ontologies) a formal relation between them, i.e., an *axiomatic* relation such as equivalence, sub- or super-class, or disjointness. An alignment between two ontologies is created by a set of mappings. Ontology matching is the process of finding mappings between classes in two ontologies. Ontology integration goes beyond identification of an ontology alignment in that two or more ontologies are merged into a single ontology that encompasses all classes in the original ontologies [14].

Phenotype classes in the HP and MP ontologies are formally defined using the Entity-Quality (EQ) pattern [4, 26]. Based on the EQ patterns, a phenotype is decomposed into an affected entity and a quality that specifies how the entity is affected. The Entity will usually be a class taken either from an anatomy ontology or a physiology ontology. For example, the phenotype class *macroglossia* (HP:0000158) describes an anatomical abnormality and is defined as equivalent to ‘has part’ some (‘increased size’ and (‘inheres in’ some tongue)and (‘has modifier’ some abnormal)), relying on the entity *tongue* (from the UBERON anatomy ontology [9]) and the quality *increased size* (from PATO) in its definition. The class *abnormality of salivation* (HP:0100755) is a physiological abnormality and is defined as equivalent to ‘has part’ some (quality and (‘inheres in’ some ‘saliva secretion’) and (‘has modifier’ some abnormal)), where *saliva secretion* is a class from the biological process branch of the Gene Ontology (GO) [10].

The general pattern for defining a phenotype class in both the HP and MP ontologies, given Entity E and Quality Q, is to declare them equivalent to ‘has part’ some (Q and ‘inheres in’ some E). In some cases, the Entity E is further constrained, e.g., by a location in which a certain process may happen. The “E” classes are generally taken either from the UBERON cross-species anatomy ontology [9] or from the GO. As the use of anatomy and physiology ontologies (UBERON and GO) is shared between MP and HP, it is possible to integrate both ontologies directly, based on the axiom patterns used to constrain their classes. However, the type of axiom pattern used in both ontologies results in a classification that is primarily based on the PATO ontology, as the Quality *Q* is the main feature that distinguishes different classes.

In the PhenomeNET ontology, we rewrite all axioms in HP and MP using a pattern-based approach that allows us to utilize axioms from anatomy and physiology ontologies and enrich the classification of phenotype classes [11, 27]. In general, we declare phenotype classes defined using an Entity E and Quality Q as equivalent to ‘has part’ some (E and has-quality some Q) and we further add grouping classes that are defined as equivalent to ‘has part’ some ((‘part of’ some E)and has-quality some Quality). For example, based on the axiom that defines *macroglossia* (HP:0000158) as equivalent to ‘has part’ some (‘increased size’ and (‘inheres in’ some tongue) and (‘has modifier’ some abnormal)), we generate two new axioms: macroglossia Equivalent To: ‘has part’ some (tongue and has-quality some ‘increased size’) as well as ‘tongue abnormality’ EquivalentTo: ‘has part’some ((‘part of’ some tongue) and has-quality some Quality). These two axioms, together with the transitivity and reflexivity of the part of’ relation, ensure that *macroglossia* becomes a subclass of *tongue abnormality*, and that all phenotypes affecting the tongue or a part of the tongue also become a subclass of *tongue abnormality*. The aim of rewriting the axioms is to base the classification of phenotype classes primarily on anatomical or physiological entities instead of the quality, and to utilize the axioms involving parthood in anatomy and physiology ontologies [11, 28]. Crucially, all axioms we generate fall in the OWL 2 EL profile [29, 30] and allow efficient automated reasoning using optimized OWL 2 EL reasoners such as ELK [31]. The first version of the PhenomeNET ontology (PhenomeNET-Plain) consists only of these axioms and no additional mappings.

In addition to this knowledge-based approach to linking the HP and MP ontologies, we also add lexical mappings, mappings derived from cross-references in the ontologies [5], mappings between HP and MP from BioPortal [22], and mappings generated by the AgreementMaker Light (AML) [14] in its default settings with a score greater than 0.7. Each mapping is added as a single equivalent classes axiom to the PhenomeNET ontology (PhenomeNET-Plain) to generate a version of the PhenomeNET ontology with lexical mappings (PhenomeNET-Map).

**Fig. 1:**
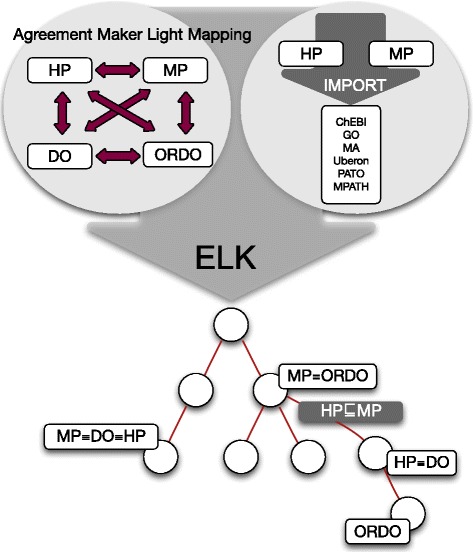
An overview of the data sources and strategies used to generate the PhenomeNET ontologies. On one side, we use mappings between HP, MP, DO, and ORDO, generated using the AML ontology matching system; on the other side, we use the axioms used to define classes in HP and MP together with the background knowledge in other ontologies to generate mappings formally. Using the ELK reasoner, we generate a hierarchical ontology structure (i.e., a taxonomy) from which we derive equivalent class, sub-class, and super-class mappings. The PhenomeNET-Full ontology is based on a combination of all these mapping approaches, while PhenomeNET-Map uses only the AML-generated mappings between HP and MP. PhenomeNET-Plain does not use any of the AML-generated mappings but solely relies on the axioms and background knowledge

Neither HP nor MP contain mappings to the DO or ORDO ontologies, despite a significant overlap between the four ontologies. Moreover, since neither DO nor ORDO contain axioms that follow a similar pattern to the axioms in HP and MP, we have to rely exclusively on lexical mappings in order to integrate DO and ORDO. To achieve this, we use the AML [14] in its default settings to generate mappings between HP and DO, HP and ORDO, MP and DO, MP and ORDO, and DO and ORDO (see Table 1). We then add an equivalent class axiom for each mapping AML identifies with a score greater than 0.7. The resulting ontology (PhenomeNET-Full) contains HP, MP, ORDO, and DO, and can be used to generate further mappings between these ontologies. Figure 1 provides an overview of the different data sources we used to generate the mappings for the three PhenomeNET ontologies.

**Table 1:** Number of classes, axioms and mappings in the PhenomeNET and AML ontologies

System	Ontology	Number of classes	Number of axioms	Mappings added
PhenomeNet-Plain	HP-MP	219,423	1,399,411	0
PhenomeNet-Map	HP-MP+mappings	219,423	1,400,570	1,160(AML), 639(BioPortal)
PhenomeNet-Full	HP-MP+DO-ORDO	241,817	1,631,543	1,489(AML), 1,018(BioPortal)
				HP-MP: 1,160 (AML),
				639(BioPortal);
				DO-MP: 423 (AML);
				DO-HP: 1,074 (AML);
				ORDO-MP: 151 (AML);
				ORDO-HP: 531 (AML);
AML	HP-MP mappings	32,509	229,337	1,160(AML)

All versions of the PhenomeNET ontology contain the classes from the HP and MP ontologies as well as the subclass axioms between named classes asserted in these ontologies. Furthermore, the PhenomeNET ontology imports the ChEBI [32] and Mouse Pathology [33] ontologies using an OWL import statement. Additionally, PhenomeNET includes all classes from the UBERON, the GO, the BioSpatial Ontology [34], the Zebrafish Anatomy ontology [35], the PATO ontology [4], the Cell Ontology [36], and the Neuro-Behavior Ontology [37]. However, these ontologies are not directly imported but rather pre-processed so that all disjointness axioms from these ontologies are excluded while all other axioms contained within them are included in the PhenomeNET ontology. The aim of this pre-processing step is to avoid unsatisfiable classes due to different conceptualizations between anatomy and phenotype ontologies, or within anatomy ontologies (Zebrafish Anatomy and UBERON) [3].

Mappings between ontologies included in PhenomeNET are generated using the ELK reasoner [31]. We use ELK to classify the PhenomeNET ontology and identify pairs of equivalent classes *C*_1_ and *C*_2_ that belong to the ontologies to be aligned. These constitute equivalent class mappings. Furthermore, we also use ELK to identify pairs of classes *C*_1_ and *C*_2_ such that *C*_1_ is a proper sub- or super-class of *C*_2_ to generate sub- and super-class mappings. A reasoner such as ELK is also required to explore and visualize the PhenomeNET ontology structures, and the PhenomeNET-Map ontology can be explored and visualized in the AberOWL ontology repository [38].

### Evaluation of mappings: HP and MP

We employ the PhenomeNET ontology primarily for integrating the HP and MP ontologies. Using the axioms in the ontology alone (PhenomeNET-Plain), we identify 745 equivalent classes between the HP and MP ontologies (see Table 2). Additionally, a large number of sub- and super-class mappings can be identified based on querying the ontology using the ELK reasoner [31] for sub- or super-classes in the two ontologies.

**Table 2:** Equivalent and sub-equivalent classes identified. Numbers in parentheses represent inferred (subclass) mappings

System	Ontology	HP-MP (≡)	HP-MP ($\sqsubseteq $)	DO-ORDO (≡)	DO-ORDO ($\sqsubseteq $)
PhenomeNET-Plain	HP-MP	745	2707 (96,278)	0	0
PhenomeNET-Map	HP-MP+mappings	1536	3999 (107,268)	0	0
PhenomeNET-Full	HP-MP+DO-ORDO	1582	4144 (112,366)	1527	4576 (16,838)

The number of pairs of equivalent classes identified increases to 1536 when adding explicit mappings derived from AML. Of these, 370 are generated by automated reasoning and are also included in AML, 791 are generated from the AML-derived equivalent classes axioms, and 375 could only be derived through the automated reasoning. For example, using the PhenomeNET ontology, we infer an equivalence class mapping between *Copper accumulation in brain* HP:0012676) and *Increased brain copper level* (MP:0011214) based on their shared definition ‘has part’ some (‘increased amount’ and (‘inheres in’ some (‘copper atom’ and (‘part of’ some brain))) and (‘has modifier’ some abnormal)). Such mappings are not easily identified by methods that do not consider the axioms constraining the ontology classes.

Additionally, we observe an increase in the number of equivalent class mappings when adding the ORDO and DO ontologies to the PhenomeNET ontology. The increase in mappings (from 1536 to 1582 classes) is a result of additional inferences obtained from adding the mappings from HP and MP to ORDO and DO, and combining them with the axioms in the PhenomeNET ontology. For example, we infer a new mapping between *decreased IgG level* (MP:0001805) and *agammaglobulinemia* (HP:0004432) based on the equivalence axioms between both classes and *agammaglobulinemia* (DOID:2583) generated by AML (based on the asserted synonym “hypogammaglobulinemia” shared between the classes in DO and MP). Table 2 summarizes our results.

### Evaluation of mappings: ORDO and DO

PhenomeNET is primarily designed for ontologies that follow the Entity-Quality definition pattern based on the PATO ontology. Neither ORDO nor DO follow this pattern, and ORDO and DO are primarily included in the PhenomeNET ontology through equivalent class axioms based on lexical mappings generated by AML. Notably, the mappings we generate are increased by including HP and MP. For example, we identify a mapping between *mandibulofacial dysostosis* (ORPHANET:155899) and *Treacher Collins syndrome* (DOID:2908), based on common AML-generated mappings to *mandibulofacial dysostosis* (HP:0005321).

### OAEI evaluation

PhenomeNET participated in the Ontology Alignment Evaluation Initiative (OAEI) 2016 challenge where several ontology alignment systems where evaluated according to the following criteria:

- precision and recall with respect to a silver standard generated by voting (using either two or three votes) the outputs of the participating systems,
- recall with respect to manually generated mappings,
- and a manual assessment of the mappings that were unique to a particular system.

In the first dataset, a silver standard reference alignment was generated from the systems participating in the OAEI challenge, using a vote of two of the participating systems. PhenomeNET-Full reached an F-measure of 0.829 in the HP-MP task and 0.886 in the DO-ORDO task. The LogMap system [39] achieved the highest F-measure in this evaluation of 0.925 for the HP-MP task, and the FCA_Map system [40] achieved the an F-measure of 0.962 in the DO-ORDO task. Results are similar when evaluating with a silver standard reference alignment generated by three votes of systems participating in the challenge. In particular in the DO-ORDO evaluation, PhenomeNET-Full achieved the second-highest F-score of 0.935, while the LogMap system [39] achieved an F-measure of 0.937.

When evaluating against manually created mappings, PhenomeNET-Full achieved the highest recall of 0.897 in the HP-MP task but could not generate any of the manually created mappings between DO and ORDO. Furthermore, when evaluating mappings that were uniquely identified by individual systems, 89 mappings between HP and MP as well as 3 mappings between ORDO and DO were generated only by the PhenomeNET ontologies and no other participating system. These were manually assessed, and PhenomeNET obtained a precision of 1.0 both for the 89 unique mappings generated between HP and MP as well as for the 3 mappings generated between DO and ORDO. We provide full evaluation for the OAEI as Additional file 1; results are also available at http://oaei.ontologymatching.org/2016/results/phenotype/.

As PhenomeNET relies on generating a taxonomic structure in which classes from HP and MP are combined, PhenomeNET also generated a large number of subclass and superclass mappings. While these were not explicitly evaluated, PhenomeNET was the only system explicitly focusing on these kind of mappings, while other participating systems primarily focused on identifying mappings represented class equivalence.

### Predicting gene–disease associations

To determine the impact of the different mapping approaches in biomedical data analysis, we also apply the three ontologies in the task for which PhenomeNET was originally designed, predicting gene–disease associations based on semantic similarity between mouse model phenotypes and human phenotypes [3, 5]. For this purpose, we use the PhenomeNET ontology as an integrated version of both HP and MP so that semantic similarity can be computed simultaneously over both ontologies. Semantic similarity establishes a measure of relatedness between classes, or sets of classes, within an ontology (or, in some cases, between classes from multiple ontologies) [25].

To evaluate the success of the three ontologies in disease gene prioritization, we obtain mouse model phenotypes associated with loss-of-function mutations in single genes from the MGI database [23] as well as human disease phenotypes associated with Mendelian diseases from the HPO database [12], and apply a semantic similarity measure [24, 41] to compare the phenotypic similarity between phenotypes associated with mouse mutants and human disease. We systematically compute phenotypic similarity between 9131 loss-of-function mouse mutants and 7066 diseases. We perform this experiment three times, once for each version of the PhenomeNET ontology (PhenomeNET-Plain, PhenomeNET-Map, and PhenomeNET-Full). Additionally, to determine the effect of PhenomeNET’s knowledge-based approach, we also generate an integrated ontology based only on an alignment between HP and MP generated by AML.

**Fig. 2:**
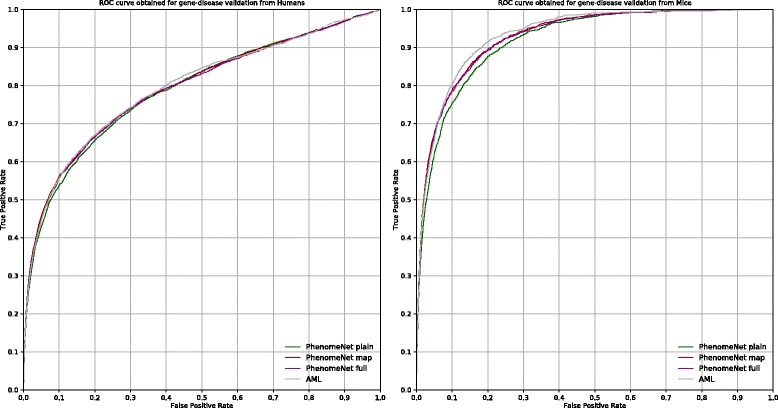
ROC curves for predicting gene–disease associations using the three different ontologies

We test how well this approach recovers known gene–disease associations. We use two sets for this evaluation: human gene–disease associations observed in a clinical context and presented in the Online Mendelian Inheritance in Man (OMIM) database [42], and mutant mice identified by curators as models of a human disease represented in the MGI database [23]. The receiver operating characteristic (ROC) curves [43] for this evaluation are shown in Fig. 2. We find that the PhenomeNET-Map version, which focuses specifically on generating mappings between MP and HP, performs best among our ontologies in this evaluation (AUROC 0.794 for human gene–disease associations and 0.930 for mouse associations), followed by PhenomeNET-Full (AUROC 0.791 for human 0.929 for mouse gene–disease associations) and PhenomeNET-Plain (AUROC 0.790 and 0.920 for human and mouse, respectively). An ontology generated only by mappings from AML, however, performs better than any of the PhenomeNET ontologies despite producing a fewer number of mappings. Using an ontology based only on the AML-derived mappings we achieve a AUROC of 0.795 and 0.934 for the human and mouse evaluation sets, respectively. However, none of the differences between the ontologies is statistically significant (*p*>0.05 for all 12 comparisons, Wilcoxon rank sum test, Bonferroni correction).

## Discussion

### Related work

A large number of ontology matching and alignment systems have been developed [44]. Several of these approaches have been applied to the same ontologies we use here. Most ontology matching systems combine methods based on lexical matching of class labels and synonyms, structural matches based on graph representations of ontologies, and background knowledge coming from a variety of sources. We make use of the AML system [14] since AML is one of the leading ontology alignment systems. AML implements a modular ontology matching framework based on lexical and structural matching methods. AML can further make use of external resources such as DO, UBERON, MeSH and Wordnet, which provide background knowledge to improve the generation of mappings, and AML further provides methods for identifying and repairing incoherent matches between two ontologies. Similarly, LogMap [39] utilizes multiple sources of background knowledge, including related ontologies from BioPortal, and utilizes lexical matching together with background knowledge to generate alignments. LogMap further uses a reasoner to identify and repair inconsistent or incoherent mappings. Similar ontology mapping systems include XMap [45] and the cross-lingual ontology matching system LYAM++ [46]. A distinct approach is FCA-Map [40], based on Formal Concept Analysis (FCA) [47], which constructs formal contexts of classes from its properties and relations, generates lattice structures and matches these concept lattices at a lexical and structural level.

One of the key features of the PhenomeNET system is its ability to identify subclass mapping in additional to equivalent class mappings. While PhenomeNET uses a reasoner for this purpose and relies on axioms that have specifically been developed for phenotype ontologies [4], alternative approaches can also identify subclass mappings based on matching sub-structures in ontology hierarchies or based on supervised machine learning [48, 49].

Due to the importance of integrating species-specific phenotype ontologies for biomedical research [50], several methods have been developed that specifically focus on the integration of phenotype ontologies. For example, PhenoHM [51] uses the Unified Medical Language System (UMLS) MetaMap service [52] to map classes from MP to UMLS concepts describing disorders and phenotypes. The Uberpheno ontology [53] as well as the Monarch knowledge graph [54] use a combination of lexical mappings and ontology axioms to generate mappings between MP and HPO. The main difference to our work lies in the representation patterns that are used to represent phenotype classes and the way in which lexical mappings are generated.

An alternative approach to finding relations between biomedical ontologies is to identify mappings between classes from different ontologies not based on the semantics of a class itself but rather based on shared annotations of the classes. For example, orthologous gene annotations can be used to identify orthologous phenotypes and thereby establish orthology relations between phenotypes. This approach has been used previously to identify yeast models for mammalian vasculature formation [55], a striking discovery since yeast has no blood vessels. To the best of our knowledge, these approaches have not yet been combined with mappings based entirely on the semantics of ontology classes and may provide a complementary source for future work on creating ontology mappings.

### Knowledge-based, structural, and lexical mappings

PhenomeNET is one of very few systems that are primarily based on automated reasoning to generate mappings and does not rely on identifying similarity between class labels, synonyms, or other associated meta-data. PhenomeNET is a system intended to match phenotypes and as such, it is not a framework that can be applied to match ontologies in general. The axiom-based approach in PhenomeNET can be applied to any ontologies that utilize PATO and the Entity-Quality definition patterns [4, 11]. In particular, PhenomeNET can not only be used to integrate MP and HPO, but also has been used to further integrate yeast, fly, worm, slime mold, and fish phenotypes [3, 56]. Furthermore, the combination of semantic matching (using automated reasoning) and lexical matching in PhenomeNET mitigates some of the limitations of using lexical approaches alone, and we demonstrate this by inferring severa; mappings between HP and MP that cannot be inferred by other ontology matching systems.

However, relying on manually-created axioms also has several limitations. In particular, the axioms are created by domain experts, and only about half the classes in MP and HP are constrained by an Entity-Quality based axiom. Furthermore, the quality of the axioms is difficult to assess, and there are distinct differences between HP and MP in how the classes are constrained. A possible solution to these challenges could be to generate phenotype ontologies fully automatically using anatomy and physiology ontologies as templates and applying the axiom patterns we use in the PhenomeNET [57] or that are used elsewhere [26].

Another limitation of PhenomeNET is the reliance on OWL 2 EL which limits the expressivity of axiom patterns. The choice is mainly due to the size of the PhenomeNET ontology and the complexity of reasoning. However, more complex axiom patterns would enable more comprehensive classification of phenotypes involving absences and abnormalities [27]; experiments with an updated ontology will likely require improvement in OWL reasoning technologies.

Relying on automated reasoning over integrated phenotype ontologies can result in incoherencies due to different conceptualizations in the integrated ontologies. We avoid the incoherencies by removing disjointness axioms when including ontologies in PhenomeNET; however, this approach does not remove but only hide the underlying problems. Generic ontology matching systems have faced similar issues for a long time, and several methods have been proposed to automatically repair incoherent ontology mappings [58–60]. However, aligning incompatible conceptualizations across multiple ontologies is not trivial and automated methods still have limitations [61] that may require human intervention. In the future, we plan to investigate whether these methods can be applied to automatically repair some of the incoherencies we identified.

Finally, one limitation of most mapping approaches is their failure to consider subtle differences in ontological categories that may be obvious to ontology developers and users but are not always reflected in the labels. This issue is particularly prevalent in the domains of phenotypes and diseases where the same label may be used to specify different ontological categories. A phenotype such as ‘agammaglobulinemia’ is an observational phenomenon related to levels of gamma-globulin in blood, while the disease ‘agammaglobulinemia’ is a more complex entity that may involve a particular etiology and several signs and symptoms (of which the phenotype ‘agammaglobulinemia’ may be one). Inspection of the written definition of ‘agammaglubulinemia’ (HP:0004432) in HPO indicates that the class refers to a deficiency or absence of immunoglobulins in serum. However, the DO defines a class with this label as an immunodeficiency syndrome that includes agammaglobulinemia as part of its phenotype, and ORDO similarly implies that this is a disease but only because of its position as a child of ‘Immunodeficiency predominantly affecting antibody production’ (as there is no textual or formal definition of the class in ORDO). In MedGen (UID:168), the UMLS code C0001768 references both to HP:0004432 and Orphanet (ORPHANET:183669), but classifies both under ‘Disease or Syndrome’.

There are similar issues with, for example, *hypoglycaemia* which occurs in DO as a child of glucose metabolism disease (DOID:9993), therefore as a disease, and in HPO (HP:0001943) as a phenotype defined as ‘A decreased concentration of glucose in the blood’. HPO cross-references the UMLS (C0020615) which uses this concept in the phenomenological sense yet again classifies it under ‘Disease or Syndrome’. The ambiguity of whether two classes are equivalent, even if they use the same label, is therefore deeply embedded in the ontologies, their structure and general domain, and there is no clear way to disambiguate these types of classes without manual expert inspection or, indirectly, by their different uses for annotation and analysis of data [62]. The semantic ambiguity reflected in these examples is partly a consequence of clinical usage of language where different entities (such as a disease and phenotype) are referred to by the same name. Resolving these issues is a problem that can likely only be addressed through expert curation across a very wide range of ontologies. The impact of these issues is likely not severe for human ontology users, but it remains a problem for any semantic approach to knowledge capture, analysis, and integration, and not least in the area of ontology matching.

## Conclusions

We have developed an ontology matching system for disease and phenotype ontologies. We generated three different version of the PhenomeNet ontology, each with different information and ontologies included. PhenomeNET is primarily based on deductive inference and automated reasoning, and while it can utilize lexically-derived mappings in the ontology generation process, it does not on its own include any lexical matching algorithms. Our results demonstrate that a combination of lexical and semantic approaches may improve upon mappings between ontologies generated using only one of these methods.

## Additional file

Additional file 1 Supplementary Information. The supplementary information contains the full evaluation results of the PhenomeNET system and other participating systems for the OAEI challenge 2016. (PDF 26 kb)

## Abbreviations

OWL: Web ontology language
