# Supplementary material for: Integrating phenotype ontologies with PhenomeNET
Source: J Biomed Semantics. 2017 Dec 19;8:58. doi: 10.1186/s13326-017-0167-4 (PMC5735523; doi:10.1186/s13326-017-0167-4)
Supplement: Additional file 1 — Supplementary Information. The supplementary information contains the full evaluation results of the PhenomeNET system and other participating systems for the OAEI challenge 2016. (PDF 26 kb) [file 13326_2017_167_MOESM1_ESM.pdf]

Supplementary information: OAEI 2016 evaluation results for the phenotype track

OM algorithm	Track task	Mappings		Precision Silver 2	Recall Silver 2	F-Score Silver 2	Sum F-Scores Silver 2	Precision Silver 3	Recall Silver 3	F-Score Silver 3	Sum F-Scores Silver 3
		Equivalent (≡)	Subclasses (⊑)								
AML	HP-MP	1755	0	0.9305	0.7998	0.8602	1.7684	0.8536	0.9446	0.8968	1.7714
AML	DOID-ORDO	2098	0	0.8532	0.9708	0.9082		0.7784	0.9981	0.8747	
DiSMatch	HP-MP	644	0	0.5481	0.2058	0.2993	0.3818	0.4550	0.1971	0.2750	0.3423
DiSMatch	DOID-ORDO	335	0	0.2269	0.0505	0.0825		0.1910	0.0408	0.0673	
FCA_Map	HP-MP	1590	0	0.9836	0.7543	0.8539	1.8162	0.9421	0.9244	0.9332	1.8706
FCA_Map	DOID-ORDO	1803	0	0.9662	0.9586	0.9624		0.8880	0.9926	0.9374	
LogMap	HP-MP	2011	0	0.9354	0.9125	0.9238	1.8372	0.7732	0.9729	0.8617	1.728
LogMap	DOID-ORDO	1667	0	0.9520	0.8779	0.9134		0.9052	0.9375	0.9211	
LogMapBio	HP-MP	2151	0	0.9182	0.9315	0.9248	1.8338	0.7545	0.9824	0.8535	1.7580
LogMapBio	DOID-ORDO	1804	0	0.9202	0.8980	0.9090		0.8642	0.9487	0.9045	
LogMapLite	HP-MP	667	0	1.0000	0.3449	0.5129	1.2284	0.9985	0.4471	0.6176	1.3814
LogMapLite	DOID-ORDO	1000	0	0.9930	0.5592	0.7155		0.9890	0.6221	0.7638	
LYAM	HP-MP	381	0	0.4068	0.0685	0.1172	0.1172	0.1654	0.0359	0.0590	0.0590
LYAM	DOID-ORDO	0	0	0.0	0.0	0.0		0.0	0.0	0.0	
PhenomeNET-Full	HP-MP	1582	4144 (112.366)	0.7568	0.9164	0.8290	1.7149	0.6292	0.9452	0.7555	1.6905
PhenomeNET-Full	DOID-ORDO	1582	4144 (112.366)	0.9498	0.8301	0.8859		0.9472	0.9233	0.9351	
PhenomeNET-Map	HP-MP	1536	3999 (107268)	0.7708	0.9051	0.8326	0.8326	0.6441	0.9389	0.7641	0.7641
PhenomeNET-Map	DOID-ORDO	0	0	0	0.0	0.0		0.0	0.0	0.0	
PhenomeNET-Plain	HP-MP	745	2.707 (96.278)	0.7828	0.5784	0.6653	0.6653	0.6391	0.5076	0.5658	0.5658
PhenomeNET-Plain	DOID-ORDO 0	0	0	0.0	0.0	0.0		0.0	0.0	0.0	
XMap	HP-MP	650	0	1.0000	0.3332	0.4998	1.12213	1.0000	0.4351	0.6064	1.3739
XMap	DOID-ORDO	1030	0	0.9845	0.5693	0.7214		0.9767	0.6320	0.7674	

Table 1: Precision, Recall, F-measure obtained against the silver standard with vote 2 and 3

OM algorithm	HP-MP	DOID-ORDO
PhenomeNET-Full	0.8966	0.0000
PhenomeNET-Map	0.8966	0.0000
PhenomeNET-Plain	0.8276	0.0000
AML	0.7586	0.0000
LogMapBio	0.6897	0.1667
LogMap	0.6552	0.1167
FCA-Map	0.6207	0.0000
LogMapLite	0.5172	0.0000
XMap	0.5172	0.0000
DiSMatch	0.1379	0.0333
LYAM	0.0000	0.0000

Table 2: Precision for manually created mappings

OM algorithm	Track task	Unique Equivalence Mappings	Precision (manual assessment)	Positive contribution (true positives)	Negative contribution (false positives)
AML	HP-MP	122	0.8667	8.63%	1.33%
DiSMatch	HP-MP	291	0.8333	19.80%	3.96%
FCA_Map	HP-MP	26	0.9615	2.04%	0.08%
LogMap	HP-MP	130	0.9330	9.90%	0.71%
LogMapLite	HP-MP	0	0.0	0.0%	0.0%
LogMapBio	HP-MP	176	0.9330	13.40%	0.96%
LYAM++	HP-MP	226	0.7000	12.91%	5.53%
PhenomeNET-Full	HP-MP	89	1.0000	7.27%	0.0%
PhenomeNET-Map	HP-MP	85	1.0000	6.94%	0.00%
PhenomeNET-Plain	HP-MP	80	1.0000	6.53%	0.00%
XMAP	HP-MP	0	0.0000	0.00%	0.00%
**Totals**	HP-MP	**1225**		**87.42**%	**12.58**%

Table 3: Evaluation of unique mappings (HP-MP task)

OM algorithm	Track task	Unique Equivalence Mappings	Precision (manual assessment)	Positive contribution (true positives)	Negative contribution (false positives)
AML	DOID-ORDO	308	0.8667	30.40%	4.68%
DisMatch	DOID-ORDO	259	0.4000	11.80%	17.70%
FCA_Map	DOID-ORDO	61	0.8330	5.79%	1.16%
LogMap	DOID-ORDO	80	0.9000	8.20%	0.91%
LogMapLite	DOID-ORDO	7	0.5000	0.40%	0.40%
LogMapBio	DOID-ORDO	144	0.9667	15.85%	0.55%
LYAM++	DOID-ORDO	0	0.0000	0.00%	0.00%
PhenomeNET-Full	DOID-ORDO	3	1.0000	0.34%	0.00%
PhenomeNET-Map	DOID-ORDO	0	0.0000	0.00%	0.00%
PhenomeNET-Plain	DOID-ORDO	0	0.0000	0.00%	0.00%
XMap	DOID-ORDO	16	0.5625	1.03%	0.80
**Totals**	HP-MP	**878**		**73.81**%	**26.19**%

Table 4: Evaluation of unique mappings (DOID-ORDO task)
